# Impact of Medical Residency Programs on Emergency Department Efficiency

**DOI:** 10.3390/medicina61060999

**Published:** 2025-05-28

**Authors:** Myeong Namgung, Sung Jin Bae, Ho Sub Chung, Kwang Yul Jung, Yun Hyung Choi, Chan Woong Kim, Ye Lim Gong, Ji Yun Lee, Dong-Hoon Lee

**Affiliations:** 1 Department of Emergency Medicine, College of Medicine, Chung-Ang University, Seoul 06973, Republic of Korea; myeong1518@cau.ac.kr (M.N.); uzimuz85@gmail.com (S.J.B.); hoshap@cau.ac.kr (H.S.C.); kyjung@cau.ac.kr (K.Y.J.); yunhyung0710@gamil.com (Y.H.C.); whenever@cau.ac.kr (C.W.K.); 2 Department of Digital Strategy, Chung-Ang University Healthcare System, Seoul 06973, Republic of Korea; 20945@cauhs.or.kr (Y.L.G.); 19784@cauhs.or.kr (J.Y.L.)

**Keywords:** medical residency program, emergency department efficiency, clinical process time

## Abstract

*Background and Objectives*: Medical residency programs play a crucial role in emergency departments (EDs). However, clinical processes may differ between EDs staffed with medical residents and those staffed only by attending physicians. This study aims to compare clinical process times and clinical outcomes between these two types of EDs. *Materials and Methods*: A retrospective observational study was conducted, analyzing patients aged ≥ 18 years who visited an ED with a medical residency program and an ED staffed only by attending physicians. Time variables, including the time to first physician encounter, first order, CT order, consultation request, consulted specialist arrival, disposition decision, ED length of stay (LOS), and ED dispositions were compared between the two ED settings. *Results*: A total of 24,942 patients in an ED with a medical residency program and 19,867 patients in an ED staffed only by attending physicians were included in the final analysis. The ED with a medical residency program exhibited significantly longer times in all time variables including time to first physician encounter (7.0 [4.0–12.0] vs. 3.0 [1.0–5.0] min), first order (15.0 [9.0–23.0] vs. 9.0 [5.0–13.0] min), consultation request (95.0 [42.0–146.0] vs. 72.0 [27.0–124.0] min), consulted specialist arrival (156.0 [90.0–238.0] vs. 117.0 [63.0–176.0] min), and disposition decision (134.0 [70.0–208.0] vs. 92.0 [32.0–139.0] min). However, the proportion of discharges, admissions, and deaths were similar between the two EDs. *Conclusions*: The ED with a medical residency program may contribute to delays in clinical processing times; however, it appears to have no significant impact on clinical outcomes.

## 1. Introduction

Emergency medicine plays a critical role in modern healthcare systems, providing immediate, life-saving care to patients with acute conditions. As the field continues to expand, medical residency programs have been developed worldwide to standardize training for emergency medicine physicians. In 2003, the Korean Society of Emergency Medicine established an emergency medicine residency program to ensure a structured and standardized training process. The revised training curriculum, updated in 2016, is currently implemented across training hospitals nationwide [1]. Consequently, in most training hospitals, patient care and treatment are primarily resident-centered as part of the training process.

However, when residents primarily manage patient care, the clinical process may differ from that in emergency departments (EDs) where attending physicians lead care. It is generally understood that physicians acquire greater knowledge and skills with experience, which may influence decision-making and patient management [2]. Differences in decision-making may arise from the time a patient arrives at the ED to the point when a discharge or admission decision is made. In ED settings, both diagnostic and treatment accuracy, as well as decision-making speed, are critical. The current emergency care environment faces persistent challenges, including shortages and imbalances in healthcare personnel and ongoing ED overcrowding. Delays in decision-making can prolong patient stays, further exacerbating congestion and negatively affect clinical outcomes [3,4,5,6].

Therefore, in this study, we compared the efficiency of an ED with a medical residency program to that staffed only by attending physicians, analyzing differences in clinical processing times and clinical outcomes. Additionally, we investigate how the absence of residents, following the February 2024 medical resident strike in South Korea, has influenced emergency department workflows.

## 2. Materials and Methods

### 2.1. Study Design and Population

We conducted a retrospective observational study of all patients older than 18 years who visited two EDs affiliated with university hospitals in Seoul. One hospital, with approximately 730 beds, originally operated a medical residency program in which attending physicians and medical residents worked together in the ED. The other hospital, a newly established facility with approximately 700 beds, followed the same system as the parent hospital but was staffed only by attending physicians, as it was not a teaching hospital. In February 2024, most medical residents in South Korea participated in a general strike opposing government policies, leaving their workplaces. During the strike period, the ED with a medical residency program in this study was staffed only by attending physicians, as no medical residents were present. Accordingly, the study period was divided into two phases: before the strike (1 March 2023 to 31 August 2023) and during the strike (1 March 2024 to 31 August 2024). This study was approved by the institutional review boards (IRB No. 2501-210-001). A retrospective review of the hospital database was conducted, and all patients were anonymized before the analysis. Therefore, the requirement for informed consent was waived by the institutional review board.

### 2.2. Data Collection

We obtained data from the National Emergency Department Information System (NEDIS), a national database system that transmits and analyzes the information of patients visiting the ED in real time, and from the electronic medical records (EMRs). The analyzed data included patient age, sex, Korea Triage and Acuity Scale (KTAS) level (Level I: resuscitation; Level II: emergency; Level III: urgent; Level IV: less urgent; and Level V: non-urgent), vital signs at presentation, disposition, and time-related variables included the time from ED arrival to the first physician encounter, time to first order, time to computed tomography (CT) order, time to consultation with other departments, time to consulted specialist arrival at the ED, time to disposition decision, and ED length of stay (LOS). For patients admitted to the intensive care unit (ICU), additional data were collected, including time from ED arrival to intubation, time to central catheter insertion, time to arterial catheter insertion, hospital LOS, and post-admission outcomes.

### 2.3. Outcome Measurement

The primary outcome was the comparison of clinical processing times at each stage between the ED with a medical residency program and the ED staffed only by attending physicians. The secondary outcome was the comparison of clinical outcomes across the same ED settings. Outcomes were analyzed across three comparisons based on the ED staffing and study periods: (1) an ED with a medical residency program versus an ED staffed only by attending physicians before the strike; (2) an ED with a medical residency program and an ED staffed only by attending physicians before and during the strike; and (3) an ED with a medical residency program versus an ED staffed only by attending physicians during the strike. Additionally, a subanalysis was conducted to compare the outcomes among critically ill patients admitted to the ICU from the EDs before and during the strike in both settings.

### 2.4. Statistical Analysis

All statistical analyses were conducted using R software (version 4.4.2). Categorical variables were expressed as percentages, and continuous variables were presented as medians with interquartile range (IQR). The chi-squared test was used to compare categorical variables, while Mann–Whitney U-test was applied to compare continuous variables. Differences were considered statistically significant at *p* < 0.05. Furthermore, patient clinical processes in both EDs were analyzed using process mining techniques for each study period. We employed ProFIT (https://github.com/itmo-escience/ProFIT, accessed on 21 February 2025), an open-source implementation of the fuzzy mining algorithm originally developed by Günther and van der Aalst [7]. To achieve an optimal balance between signal detection and noise suppression in the data, we set the “set_rates()” parameter to 90 for both activities and paths. Records with missing time-stamp data essential for event log construction were excluded to ensure the accuracy of the process mining analysis. Workflow optimization was performed, and the time at each step was represented by the median value. To visually emphasize the differences in clinical process times at each stage, the median values were normalized within a range of 0 to 100 min and mapped to a colormap.

## 3. Results

**Figure 1:**
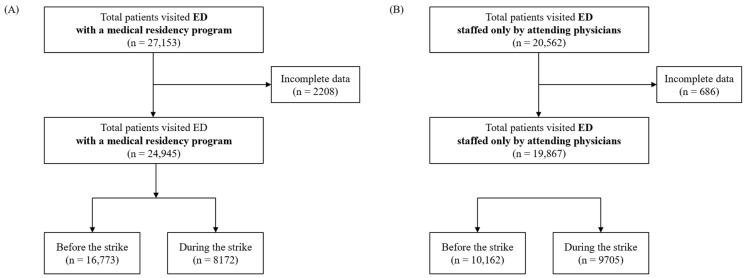
Flow chart of patients enrolled in the study. (**A**) ED with a medical residency program; (**B**) ED staffed only by attending physicians.

The number of patients visiting the ED with a medical residency program was 16,773 before the strike and 8172 after the strike, representing a 51.3% decrease (Figure 1A). In contrast, the number of patients visiting the ED staffed only by attending physicians was 10,162 before the strike and 9705 during the strike, reflecting a 4.5% decrease (Figure 1B).

### 3.1. ED with a Medical Residency Program Versus ED Staffed Only by Attending Physicians Before the Strike

**Figure 2:**
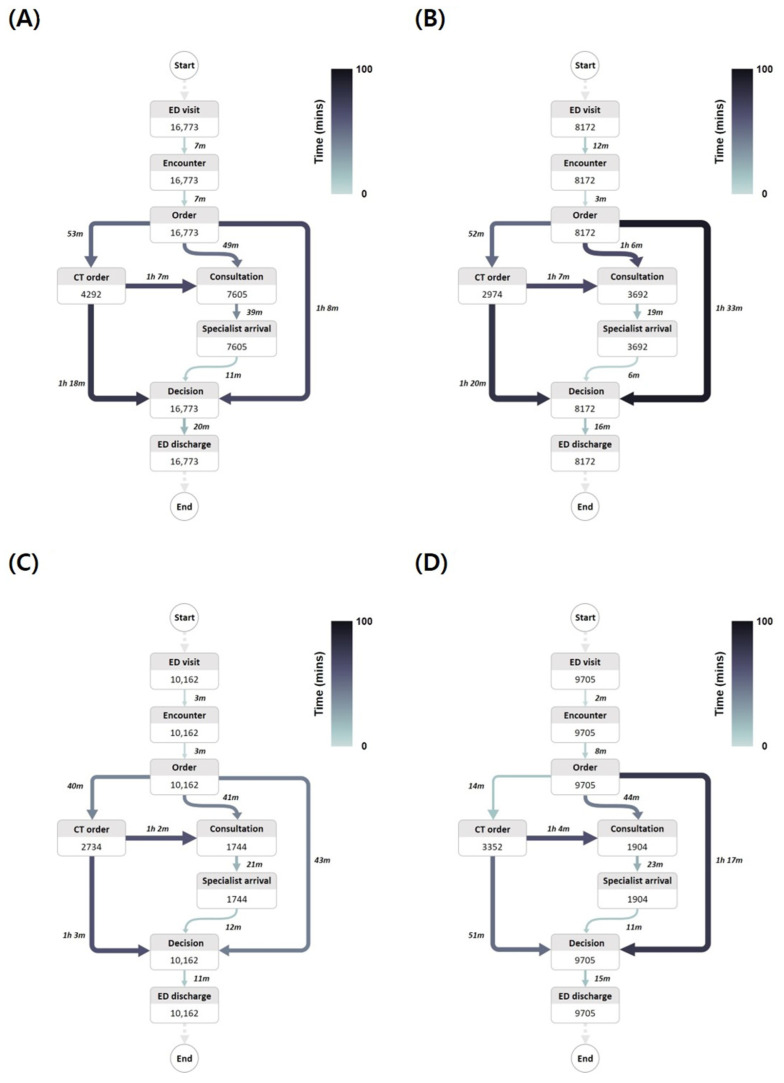
Process mining of clinical workflow in both EDs. (**A**) ED with a medical residency program before the strike; (**B**) ED with a medical residency program during the strike; (**C**) ED staffed only by attending physicians before the strike; (**D**) ED staffed only by attending physicians during the strike.

All time variables were significantly longer in the ED with a medical residency program compared to the ED staffed only by attending physicians (Table 1, Figure 2A,C). The time to disposition decision (134.0 [70.0–208.0] min vs. 92.0 [32.0–139.0] min, *p* < 0.001) and ED LOS (165.0 [95.0–260.0] min vs. 116.0 [49.0–174.0] min, *p* < 0.001) were particularly prolonged in the ED with a residency program. The proportion of first (45.3% vs. 17.2%, *p* < 0.001) and second (4.0% vs. 0.6%, *p* < 0.001) consultations was also higher in the ED with a medical residency program. While third or fourth consultations occurred in the ED with a medical residency program, no such consultations took place in the ED staffed only by attending physicians. Both EDs exhibited similar patient disposition patterns, with comparable proportions of discharge (62.0% vs. 63.0%), general ward admission (18.3% vs. 18.3%), ICU admission (3.3% vs. 3.3%), transfer to other hospitals (11.6% vs. 14.7%), and death in the ED (0.2% vs. 0.2%), except for against discharge (4.5% vs. 0.5%).

### 3.2. Before Versus During the Strike in the Two EDs

The proportion of patients with KTAS scores of 1 or 2 increased in both EDs during the strike (Table 2). In the ED with a medical residency program, during the strike, the time to CT order and the time to first consulted specialist arrival decreased, whereas the time to first physician encounter, time to first order, and time to first consultation increased (Figure 2A,B). The median time to disposition decision was identical before and during the strike (134.0 [70.0–208.0] min vs. 134.0 [90.0–188.0] min), although the difference was statistically significant (*p* = 0.011). In the ED staffed only by attending physicians, during the strike, the time to first order and the time to CT order decreased, whereas the time to first order increased (Figure 2C,D). The median time to disposition decision increased during the strike (from 92.0 [32.0–139.0] min to 108.0 [64.0–151.0] min, *p* < 0.001). During the strike, the proportion of admissions increased for both general ward admissions (from 18.3% to 25.1% in the ED with a medical residency program and from 18.3% to 21.9% in the ED staffed only by attending physicians) and ICU admissions (from 3.3% to 6.3% and from 3.3% to 4.2%, respectively).

### 3.3. ED with a Medical Residency Program Versus ED Staffed Only by Attending Physicians During the Strike

During the strike, all time variables remained longer in the ED with a medical residency program (Table 3, Figure 2B,D). However, compared to before the strike, the median difference in the time to disposition decision and ED LOS between the two EDs decreased from 42.0 to 26.0 min and 49.0 to 29.0 min, respectively. Disposition patterns remained comparable between both EDs.

### 3.4. Patients Who Were Admitted to the ICU

Both before and during the strike, the time to first physician encounter, time to CT order, time to disposition decision, ED LOS, and hospital LOS were significantly longer in the ED with a medical residency program (Table 4). In contrast, the time to central catheter insertion and time to arterial catheter insertion were longer in the ED staffed only by attending physicians. The proportions of intubation, central catheter insertion, and arterial catheter insertion were also higher in the ED with a medical residency program. In-hospital mortality was significantly higher in the ED with a medical residency program before the strike (15.2% vs. 8.0%, *p* = 0.002). However, during the strike, no significant difference was observed (15.5% vs. 11.9%, *p* = 0.136). 

### 3.5. Clinical Flow in Both EDs Before and During Strike

Figure 2 illustrates the clinical workflows in both EDs. In the ED with a medical residency program, delays in the clinical process occurred at different stages. Before the strike, delays were observed between the first order and the disposition decision, between the CT order and the disposition decision, and between the CT order and the consultation (Figure 2A). During the strike, delays were primarily noted between the first order and the consultation (Figure 2B). In the ED staffed only by attending physicians, the delayed stages also varied. Before the strike, delays were observed between the CT order and the disposition decision and between the CT order and the consultation (Figure 2C). During the strike, delays occurred between the CT order and the consultation and between the first order and the disposition decision (Figure 2D).

## 4. Discussion

In this study, we examined the effect of medical residents on ED efficiency by comparing clinical process times and clinical outcomes between an ED with a medical residency program and an ED staffed only by attending physicians. In the ED with a medical residency program, all time variables were significantly longer than those in the ED staffed only by attending physicians. Additionally, the proportion of consultations and the number of consultations were also higher. These increases in clinical processing times and stages contributed to a prolonged time to disposition decision.

Our findings are consistent with previous studies showing that the presence of residents is associated with longer door-to-order or door-to-disposition times, as well as increased ED LOS, thereby decreasing efficiency [8,9,10]. However, several studies have reported conflicting results. McGarry et al. examined the impact of adding emergency medicine residents and found no significant difference in ED LOS and admission rate [11]. Another study similarly reported that ED LOS was unaffected by the presence or total number of trainees [12]. These discrepancies are likely due to differences in trainees’ seniority levels. The studies reporting no significant impact included only participants with relatively lower seniority, such as postgraduate year one (PGY-1) residents, medical students, and interns. Since these trainees are not yet responsible for independent clinical tasks and primarily function as support staff, it is reasonable to assume that their presence has minimal impact on clinical processing times.

The finding that the clinical processing times were longer in the ED with a medical residency program than in the ED staffed only by attending physicians may be attributed to the resident-led nature of patient care, which often involves additional steps such as case discussions and supervision by attending physicians. A qualitative study by Farnan et al. on general medicine residents found that residents followed a stepwise consultation process in uncertain situations, first consulting peers and senior residents, then consulting fellows, and finally seeking guidance from attending physicians [13]. Additionally, residents may seek the opinions of specialists in other departments to reduce uncertainty. Furthermore, the process may involve a reevaluation by specialists after the initial assessment by residents. Previous studies have reported that prolonged ED LOS is associated with the number of consultations and the time from ED visit to consultation request [14,15]. These delays could ultimately contribute to longer clinical processing times and increased ED LOS. In contrast, the ED staffed only by attending physicians may have facilitated more independent decision-making. To address these challenges, implementing structured supervision models, standardized clinical protocols, and streamlining communication between residents and supervising physicians may enhance efficiency without compromising educational quality.

This contrast became more evident during the medical resident strike. In South Korea, the medical residents went on strike in response to government policies, leading to their withdrawal from the ED. In the absence of residents, significant changes were observed in the ED with a medical residency program during the strike. As time variables such as the time to CT order and time to consulted specialist arrival decreased in the ED with a medical residency program, the median differences in the time from patient arrival at the ED to the disposition decision between the two EDs decreased during the strike compared to the pre-strike period. Additionally, the admission rate to both general wards and ICUs increased in both EDs during the strike. However, no significant difference in the proportion of deaths in the ED was observed between the two periods. This finding aligns with previous studies examining the impact of resident strikes, which have reported reductions in decision-making time and ED LOS without a significant effect on mortality rates [8,16,17,18].

We also found that the absence of residents led to decrease in the number of patients visiting the EDs. Specifically, the ED with a medical residency program experienced a nearly 50% reduction in patient visits, whereas the ED staffed only by attending physicians experienced only a 4.5% decrease. This finding suggests that the absence of residents substantially impacts the ED’s capacity to accommodate patients. Productivity, defined as the number of patients seen by emergency physicians per hour, increased when residents were present [19,20].

The analysis of critically ill patients admitted to the ICU also revealed differences in workflow between the ED with a medical residency program and the ED staffed only by attending physicians. Before the strike, nearly all clinical processing time variables were significantly prolonged in the ED with a medical residency program. Although these time variables remained longer in the ED with a medical residency program during the strike, the median differences between the two EDs were markedly reduced. Furthermore, in-hospital mortality was higher in the ED with a medical residency program before the strike, although this difference was not statistically significant. The apparent difference in mortality rates was likely to due to an increase in mortality in the ED staffed only by attending physicians during the strike. The in-hospital mortality rates in the ED with a medical residency program before and during the strike were 15.2% and 15.5%, respectively, with no significant difference (*p* = 0.939) (Table A1). These findings suggest that the absence of residents did not have a significant impact on patient safety.

This study had a few limitations. First, it was conducted at two university-affiliated hospitals in South Korea, which may limit the generalizability of the findings to other healthcare settings. Second, as a retrospective study, it is susceptible to biases related to documentation and data accuracy. Third, the study period was relatively short, and the long-term effects of the resident strike on emergency care remain unknown. Fourth, the clinical efficiency of medical residents, depending on their training level (e.g., junior vs. senior residents), was not accounted for in our analysis. Fifth, we also acknowledge that no statistical adjustments were made for potential confounding variables such as patient age, triage level, or case complexity, all of which may influence clinical processing times and outcomes. Finally, although process mining techniques were used to analyze the clinical workflows, unmeasured confounding factors such as individual physician practice patterns and hospital policies may have influenced the results.

## 5. Conclusions

This study highlights the role of medical residents in ED efficiency. Although EDs with a medical residency program may be associated with prolonged clinical processing times, they do not appear to substantially affect clinical outcomes.

## Figures and Tables

**Table 1:** Comparison of time variables and clinical outcomes between the two emergency departments before the strike.

Variable	ED with a Medical Residency Program (*n* = 16,773)	ED Staffed Only by Attending Physicians (*n* = 10,162)	*p*-Value
Age (years)	53.0 (32.0–70.0)	49.0 (35.0–65.0)	<0.001
Sex (male)	7298 (43.5)	4191 (41.2)	<0.001
Triage level			<0.001
Levels 1 and 2	453 (2.7)	597 (5.9)	
Levels 3 to 5	16,320 (97.3)	9565 (94.1)	
Door to first encounter (min)	7.0 (4.0–12.0)	3.0 (1.0–5.0)	<0.001
Door to order (min)	15.0 (9.0–23.0)	9.0 (5.0–13.0)	<0.001
CT order	4292 (25.6)	2734 (26.9)	0.018
Door to CT order (min)	73.5 (23.0–115.0)	59.0 (15.0–98.0)	<0.001
1st consultation	7605 (45.3)	1744 (17.2)	<0.001
Door to consultation (min)	95.0 (42.0–146.0)	72.0 (27.0–124.0)	<0.001
Door to specialist arrival (min)	156.0 (90.0–238.0)	117.0 (63.0–176.0)	<0.001
2nd consultation	668 (4.0)	56 (0.6)	<0.001
Door to consultation (min)	183.5 (126.0–251.5)	148.5 (88.0–201.5)	0.005
Door to specialist arrival (min)	283.0 (201.0–398.5)	206.0 (143.5–335.0)	0.004
3rd consultation	59 (0.4)	0	
Door to consultation (min)	202 (143.5–307.5)	0	
Door to specialist arrival (min)	329 (228.0–510.5)	0	
4th consultation	11 (0.1)	0	
Door to consultation (min)	298 (197–346.5)	0	
Door to specialist arrival (min)	396 (370.5–490.0)	0	
Door to decision (min)	134.0 (70.0–208.0)	92.0 (32.0–139.0)	<0.001
ED length of stay (min)	165.0 (95.0–260.0)	116.0 (49.0–174.0)	<0.001
Disposition			<0.001
Discharge	10,410 (62.0)	6405 (63.0)	
Admission to general ward	3064 (18.3)	1857 (18.3)	
Admission to ICU	560 (3.3)	338 (3.3)	
Transfer	1947 (11.6)	1492 (14.7)	
Death	42 (0.2)	21 (0.2)	
Against discharge	750 (4.5)	49 (0.5)	

Categorical data are presented as numbers (%), and continuous data are presented as medians (interquartile range: Q1–Q3). KTAS: Korea Triage and Acuity Scale; ED: emergency department; ICU: intensive care unit.

**Table 2:** Comparison of time variables and clinical outcomes in the two emergency departments before and during the strike.

	ED with a Medical Residency Program			ED Staffed Only by Attending Physicians		
Variable	Before the Strike (*n* = 16,773)	During the Strike (*n* = 8172)	*p*-Value	Before the Strike (*n* = 10,162)	During the Strike (*n* = 9705)	*p*-Value
Age (years)	53.0 (32.0–70.0)	60.0 (37.0–76.0)	<0.001	49.0 (35.0–65.0)	53.0 (36.0–68.0)	<0.001
Sex (male)	7298 (43.5)	3816 (46.7)	<0.001	4191 (41.2)	4239 (43.7)	<0.001
Triage level			<0.001			<0.001
Levels 1 and 2	453 (2.7)	506 (6.2)		597 (5.9)	884 (9.1)	
Levels 3 to 5	16,320 (97.3)	7666 (93.8)		9565 (94.1)	8821 (90.9)	
Door to first encounter (min)	7.0 (4.0–12.0)	14.0 (8.0–26.0)	<0.001	3.0 (1.0–5.0)	2.0 (1.0–4.0)	<0.001
Door to order (min)	15.0 (9.0–23.0)	16.0 (10.0–25.0)	<0.001	9.0 (5.0–13.0)	11.0 (7.0–15.0)	<0.001
CT order	4292 (25.6)	2974 (36.4)	<0.001	2734 (26.9)	3352 (34.5)	<0.001
Door to CT order (min)	73.5 (23.0–115.0)	45.0 (18.0–96.0)	<0.001	59.0 (15.0–98.0)	44.0 (13.0–98.0)	0.001
1st consultation	7605 (45.3)	3692 (45.2)	<0.001	1744 (17.2)	1904 (19.6)	<0.001
Door to consultation (min)	95.0 (42.0–146.0)	102.0 (54.0–148.0)	<0.001	72.0 (27.0–124.0)	70.0 (25.0–122.0)	0.237
Door to specialist arrival (min)	156.0 (90.0–238.0)	132.0 (84.0–185.0)	<0.001	117.0 (63.0–176.0)	118.0 (66.0–179.0)	0.591
Door to decision (min)	134.0 (70.0–208.0)	134.0 (90.0–188.0)	0.011	92.0 (32.0–139.0)	108.0 (64.0–151.0)	<0.001
ED length of stay (min)	165.0 (95.0–260.0)	162.0 (114.0–224.0)	0.364	116.0 (49.0–174.0)	133.0 (90.0–191.0)	<0.001
Disposition			<0.001			<0.001
Discharge	10,410 (62.0)	5316 (65.0)		6405 (63.0)	7005 (72.2)	
Admission to general ward	3064 (18.3)	2055 (25.1)		1857 (18.3)	2130 (21.9)	
Admission to ICU	560 (3.3)	515 (6.3)		338 (3.3)	404 (4.2)	
Transfer	1947 (11.6)	118 (1.4)		1492 (14.7)	91 (0.9)	
Death	42 (0.2)	25 (0.3)		21 (0.2)	27 (0.3)	
Against discharge	750 (4.5)	143 (1.7)		49 (0.5)	48 (0.5)	

Categorical data are presented as numbers (%), and continuous data are presented as medians (interquartile range: Q1–Q3). KTAS: Korea Triage and Acuity Scale; ED: emergency department; ICU: intensive care unit.

**Table 3:** Comparison of time variables and clinical outcomes between the two emergency departments during the strike.

Variable	ED with a Medical Residency Program (*n* = 8172)	ED Staffed Only by Attending Physicians (*n* = 9705)	*p*-Value
Age (years)	60.0 (37.0–76.0)	53.0 (36.0–68.0)	<0.001
Sex (male)	3816 (46.7)	4239 (43.7)	<0.001
Triage level			<0.001
Levels 1 and 2	506 (6.2)	884 (9.1)	
Levels 3 to 5	7666 (93.8)	8821 (90.9)	
Door to first encounter (min)	14.0 (8.0–26.0)	2.0 (1.0–4.0)	<0.001
Door to order (min)	16.0 (10.0–25.0)	11.0 (7.0–15.0)	<0.001
CT order	2974 (36.4)	3352 (34.5)	0.01
Door to CT order (min)	45.0 (18.0–96.0)	44.0 (13.0–98.0)	<0.001
1st consultation	3692 (45.2)	1904 (19.6)	<0.001
Door to consultation (min)	102.0 (54.0–148.0)	70.0 (25.0–122.0)	<0.001
Door to specialist arrival (min)	132.0 (84.0–185.0)	118.0 (66.0–179.0)	<0.001
Door to decision (min)	134.0 (90.0–188.0)	108.0 (64.0–151.0)	<0.001
ED length of stay (min)	162.0 (114.0–224.0)	133.0 (90.0–191.0)	<0.001
Disposition			<0.001
Discharge	5316 (65.0)	7005 (72.2)	
Admission to general ward	2055 (25.1)	2130 (21.9)	
Admission to ICU	515 (6.3)	404 (4.2)	
Transfer	118 (1.4)	91 (0.9)	
Death	25 (0.3)	27 (0.3)	
Against discharge	143 (1.7)	48 (0.5)	

Categorical data are presented as numbers (%), and continuous data are presented as medians (interquartile range: Q1–Q3). KTAS: Korea Triage and Acuity Scale; ED: emergency department; ICU: intensive care unit.

**Table 4:** Comparison of time variables and clinical outcomes in ICU-admitted patients by two emergency departments before and during the strike.

	Before the Strike				During the Strike	
Variable	ED with a Medical Residency Program (*n* = 560)	ED Staffed Only by Attending Physicians (*n* = 338)	*p*-Value	ED with a Medical Residency Program (*n* = 515)	ED Staffed Only by Attending Physicians (*n* = 404)	*p*-Value
Door to first encounter (min)	5.0 (2.0–9.0)	2.0 (1.0–4.0)	<0.001	10.0 (5.0–21.0)	2.0 (1.0–3.0)	<0.001
Door to order (min)	8.0 (3.0–13.0)	7.0 (3.0–12.0)	0.146	9.0 (5.0–15.0)	8.0 (5.0–11.0)	<0.001
CT order	282 (50.4)	135 (39.9)	0.003	213 (41.4)	203 (50.2)	0.009
Door to CT order (min)	74.0 (27.0–111.0)	43.0 (15.0–86.5)	<0.001	60.0 (23.0–91.0)	35.0 (9.0–92.0)	0.003
Intubation	108 (19.3)	27 (8.0)	<0.001	101 (19.6)	52 (12.9)	0.008
Door to intubation (min)	60.0 (26.0–143.5)	52.0 (27.0–172.0)	0.867	38.0 (19.0–154.0)	64.5 (28.5–165.0)	0.189
Central catheter insertion	190 (33.9)	41 (12.1)	<0.001	158 (30.7)	56 (13.9)	<0.001
Door to central catheter insertion (min)	80.5 (37.0–203.0)	212.0 (98.0–363.0)	<0.001	138.0 (36.0–246.0)	223.0 (120.5–311.0)	0.001
Arterial catheter insertion	360 (64.3)	119 (35.2)	<0.001	340 (66.0)	213 (52.0)	<0.001
Door to arterial catheter insertion (min)	98.0 (37.5–294.0)	218.0 (73.0–363.0)	0.001	140.5 (29.0–309.5)	174.0 (70.0–316.0)	0.014
Door to decision (min)	171.5 (83.0–280.0)	108.0 (64.0–176.0)	<0.001	117.0 (66.0–175.5)	100.0 (52.0–167.5)	0.011
ED length of stay (min)	247.5 (151.5–351.5)	189.5 (123.0–257.0)	<0.001	175.0 (117.5–238.0)	157.0 (97.0–232.5)	0.014
Hospital length of stay (d)	12.0 (5.0–31.0)	8.0 (4.0–16.0)	<0.001	12.0 (6.0–25.0)	10.0 (5.0–20.0)	0.003
In-hospital mortality	85 (15.2)	27 (8.0)	0.002	80 (15.5)	48 (11.9)	0.136

Categorical data are presented as numbers (%), and continuous data are presented as medians (interquartile range: Q1–Q3). KTAS: Korea Triage and Acuity Scale; ED: emergency department; ICU: intensive care unit.

## Data Availability

The data presented in this study are available on request from the corresponding author. The data are not publicly available due to privacy or ethical restrictions.

## Appendix A

**Table A1:** Comparison of time variables and clinical outcomes in ICU-admitted patients in both EDs before and during the strike.

	ED with a Medical Residents			ED Staffed Only by Attending Physicians		
Variable	Before the Strike (n = 560)	During the Strike (n = 515)	*p*-Value	Before the Strike (n = 338)	During the Strike (n = 404)	*p*-Value
Door to first encounter (min)	5.0 (2.0–9.0)	10.0 (5.0–21.0)	<0.001	2.0 (1.0–4.0)	2.0 (1.0–3.0)	<0.001
Door to order (min)	8.0 (3.0–13.0)	9.0 (5.0–15.0)	<0.001	7.0 (3.0–12.0)	8.0 (5.0–11.0)	0.270
CT order	282 (50.4)	213 (41.4)	0.004	135 (39.9)	203 (50.2)	0.006
Door to CT order (min)	74.0 (27.0–111.0)	60.0 (23.0–91.0)	0.007	43.0 (15.0–86.5)	35.0 (9.0–92.0)	0.303
Intubation	108 (19.3)	101 (19.6)	0.954	27 (8.0)	52 (12.9)	0.043
Door to intubation (min)	60.0 (26.0–143.5)	38.0 (19.0–154.0)	0.118	52.0 (27.0–172.0)	64.5 (28.5–165.0)	0.984
Central catheter insertion	190 (33.9)	158 (30.7)	0.284	41 (12.1)	56 (13.9)	0.557
Door to central catheter insertion (min)	80.5 (37.0–203.0)	138.0 (36.0–246.0)	0.073	212.0 (98.0–363.0)	223.0 (120.5–311.0)	0.962
Arterial catheter insertion	360 (64.3)	340 (66.0)	0.595	119 (35.2)	213 (52.7)	<0.001
Door to arterial catheter insertion (min)	98.0 (37.5–294.0)	140.5 (29.0–309.5)	0.922	218.0 (73.0–363.0)	174.0 (70.0–316.0)	0.282
Door to decision (min)	171.5 (83.0–280.0)	117.0 (66.0–175.5)	<0.001	108.0 (64.0–176.0)	100.0 (52.0–167.5)	0.279
ED length of stay (min)	247.5 (151.5–351.5)	175.0 (117.5–238.0)	<0.001	189.5 (123.0–257.0)	157.0 (97.0–232.5)	0.001
Hospital length of stay (d)	12.0 (5.0–31.0)	12.0 (6.0–25.0)	0.855	8.0 (4.0–16.0)	10.0 (5.0–20.0)	0.016
In-hospital mortality	85 (15.2)	80 (15.5)	0.939	27 (8.0)	48 (11.9)	0.103

Categorical data are presented as numbers (%), and continuous data are presented as medians (interquartile range: Q1–Q3). ED: emergency department.
